# Metamaterials for Wireless Power Transfer: A New Open Special Issue in Materials

**DOI:** 10.3390/ma15207230

**Published:** 2022-10-17

**Authors:** Long Li, Minghai Liu, Yunhui Li, Cancan Rong

**Affiliations:** 1 Key Laboratory of High-Speed Circuit Design and EMC of Ministry of Education, School of Electronic Engineering, Xidian University, Xi’an 710071, China; 2 School of Electrical and Electronic Engineering, Huazhong University of Science and Technology, Wuhan 430074, China; 3 School of Physical Science and Engineering, Tongji University, Shanghai 200092, China; 4 School of Electrical and Power Engineering, China University of Mining and Technology, Xuzhou 221008, China

*Metamaterials for Wireless Power Transfer* is a new open Special Issue of *Materials*, which aims to publish original and review papers on new scientific and applied research and make great contributions to the finding and understanding of the use of metamaterials for wireless power transfer (WPT) and related fundamentals, characterization, and applications. 

WPT technology is becoming an important topic for the wireless industry [1], which has attracted a significant amount of attention due to its convenience, reliability, and safety. However, although the WPT technology has numerous advantages, the transfer efficiency in WPT systems decreases rapidly as the distance increases, and large electromagnetic field (EMF) noise is inevitably generated in WPT charging systems and potentially harmful to electronic components, even to human health. Metamaterials are artificially engineered composite materials or structures that exhibit special properties that are not found in nature. Metamaterials can be used to manipulate electromagnetic fields and waves, bringing entirely new physical phenomena and applications. The application of the metamaterials in WPT systems has received a great deal of attention in improving the transfer efficiency and reducing the leakage of EMF in recent years [2]. Simultaneous wireless information and power transfer (SWIPT) seems to be an attractive solution for future 6G wireless communication and IoT applications, and reconfigurable intelligent surfaces (RIS) may also be linked to metamaterials for WPT in the future. 

The research interest for the Special Issue *Metamaterials for Wireless Power Transfer* includes, but is not limited to, the following: low-frequency metamaterial-based WPT systems, electromagnetic compatibility in metamaterial-based WPT systems, wireless energy harvesting (WEH), theoretical research for metamaterial-based WPT systems, magneto-inductive and resonant waves in WPT systems via metamaterials, programmable metamaterials and metasurfaces for WPT systems, and SWIPT systems based on information metamaterials and metasurfaces.
